# Crosstalk Between Epithelial Cells, Neurons and Immune Mediators in HSV-1 Skin Infection

**DOI:** 10.3389/fimmu.2021.662234

**Published:** 2021-05-03

**Authors:** Luisa F. Duarte, Antonia Reyes, Mónica A. Farías, Claudia A. Riedel, Susan M. Bueno, Alexis M. Kalergis, Pablo A. González

**Affiliations:** ^1^ Millennium Institute on Immunology and Immunotherapy, Pontificia Universidad Católica de Chile, Santiago, Chile; ^2^ Departamento de Genética Molecular y Microbiología, Facultad de Ciencias Biológicas, Pontificia Universidad Católica de Chile, Santiago, Chile; ^3^ Departamento de Ciencias Biológicas, Facultad de Ciencias de la Vida, Universidad Andrés Bello, Santiago, Chile; ^4^ Departamento de Endocrinología, Facultad de Medicina, Escuela de Medicina, Pontificia Universidad Católica de Chile, Santiago, Chile

**Keywords:** HSV-1, immune system, nervous system, neuropeptides, skin

## Abstract

Herpes simplex virus type 1 (HSV-1) infection is highly prevalent in humans, with approximately two-thirds of the world population living with this virus. However, only a fraction of those carrying HSV-1, which elicits lifelong infections, are symptomatic. HSV-1 mainly causes lesions in the skin and mucosae but reaches the termini of sensory neurons innervating these tissues and travels in a retrograde manner to the neuron cell body where it establishes persistent infection and remains in a latent state until reactivated by different stimuli. When productive reactivations occur, the virus travels back along axons to the primary infection site, where new rounds of replication are initiated in the skin, in recurrent or secondary infections. During this process, new neuron infections occur. Noteworthy, the mechanisms underlying viral reactivations and the exit of latency are somewhat poorly understood and may be regulated by a crosstalk between the infected neurons and components of the immune system. Here, we review and discuss the immune responses that occur at the skin during primary and recurrent infections by HSV-1, as well as at the interphase of latently-infected neurons. Moreover, we discuss the implications of neuronal signals over the priming and migration of immune cells in the context of HSV-1 infection.

## Introduction

HSV-1 is an enveloped virus with a linear double-stranded DNA genome that encodes over 80 genes and belongs to the *Herperviridae* family. Importantly, this virus elicits lifelong infections by remaining latent in neurons from which sporadic viral reactivations may occur (1, 2). HSV-1 is acquired early in life and causes a broad spectrum of clinical manifestations that range from uncomplicated or mild oral and facial lesions, to life-threatening pathologies (1). Importantly, this virus is the leading cause of infectious blindness in developed countries, as well as acute viral encephalitis in adults (3).

**Figure 1:**
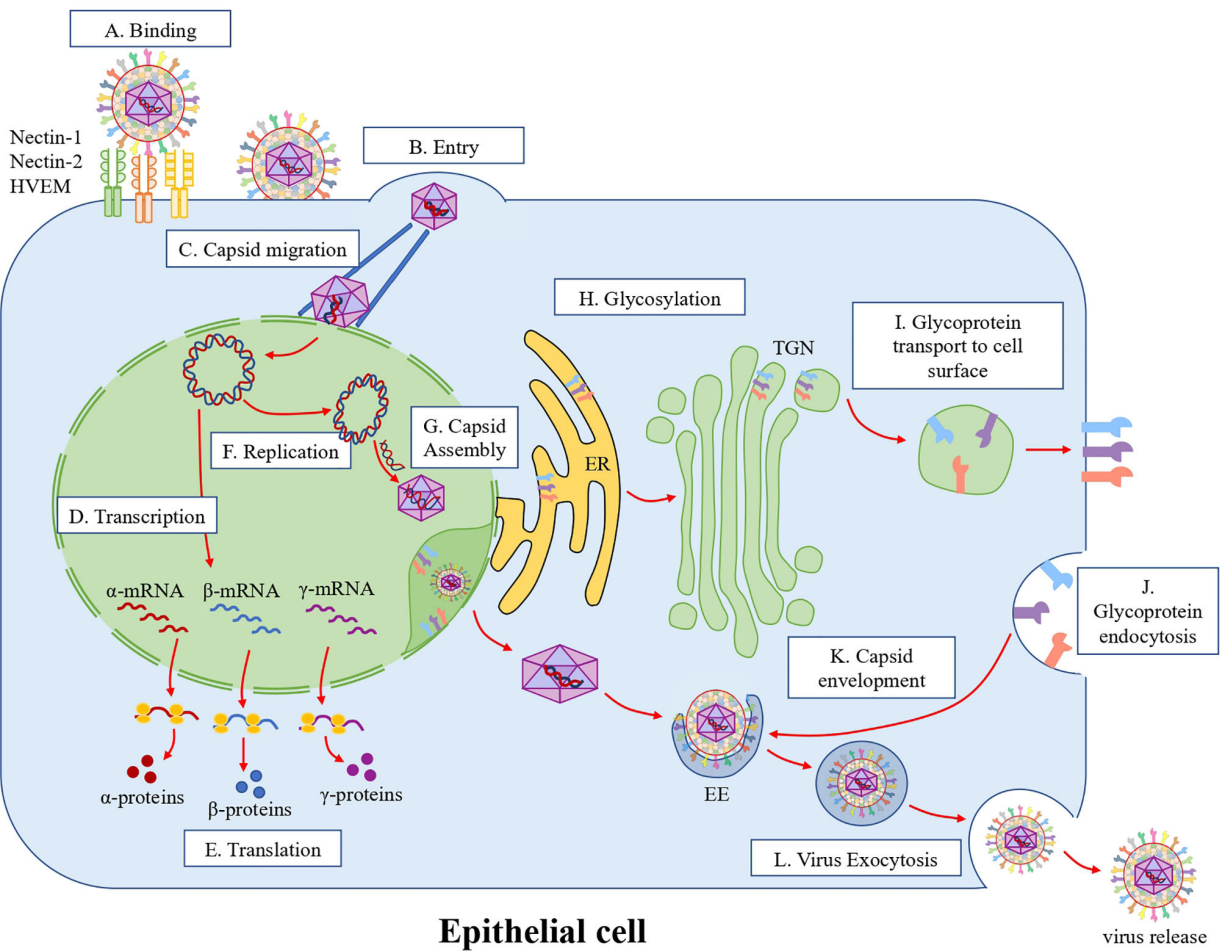
HSV-1 replication cycle in epithelial cells. **(A)** Attachment and infection. HSV-1 attaches to cell surface receptors. Glycoproteins gB and gC recognize and bind to heparan sulfate proteoglycans (HSPGs) on the cell surface. Then, gD binds to nectin-1, nectin-2 or HVEM, which activates the gH/gL complex on the virion surface, activating in turn gB that acts as a fusion protein combining the viral and cellular membranes. **(B)** Entry. After the membrane fusion event, the viral capsid and tegument proteins are released into the cytoplasm. **(C)** Capsid migration. The viral capsids are then transported through microtubules to the outer nuclear membrane, where the viral dsDNA genome is injected into the nucleus through nuclear pores. **(D)** Transcription. In the nucleus, viral alpha, beta and gamma genes are transcribed sequentially. **(E)** Translation. Viral mRNAs are then exported from the nucleus to the cytoplasm for their translation and viral protein synthesis. **(F)** Replication. The HSV-1 genome is circularized in the nucleus and is replicated as a rolling-circle. **(G)** Capsid assembly. Capsid proteins translocate into the nucleus and assemble the viral capsid in this compartment, where the viral genome is inserted into the capsid. This capsid then crosses the nuclear membrane through envelopment/de-envelopment processes. **(H)** Glycosylation. Viral glycoproteins are synthesized and initially glycosylated in the rough endoplasmic reticulum (RER). Then, glycoproteins are processed in the trans-Golgi network (TGN). **(I)** Glycoprotein transport to the cells surface. Then glycoproteins are exported to the cell surface through multivesicular bodies (MVB). **(J)** Glycoprotein endocytosis. The exported glycoproteins are endocytosed to produce viral particle envelopes in the cytoplasm. **(K)** Envelopment. Glycoproteins are concentrated in early endosomes (EE) that fuse with the viral capsid in the cytoplasm. **(L)** Virus exocytosis. Once the capsid is coated with the viral glycoproteins, the virions are released into the extracellular space through exocytosis.

**Figure 2:**
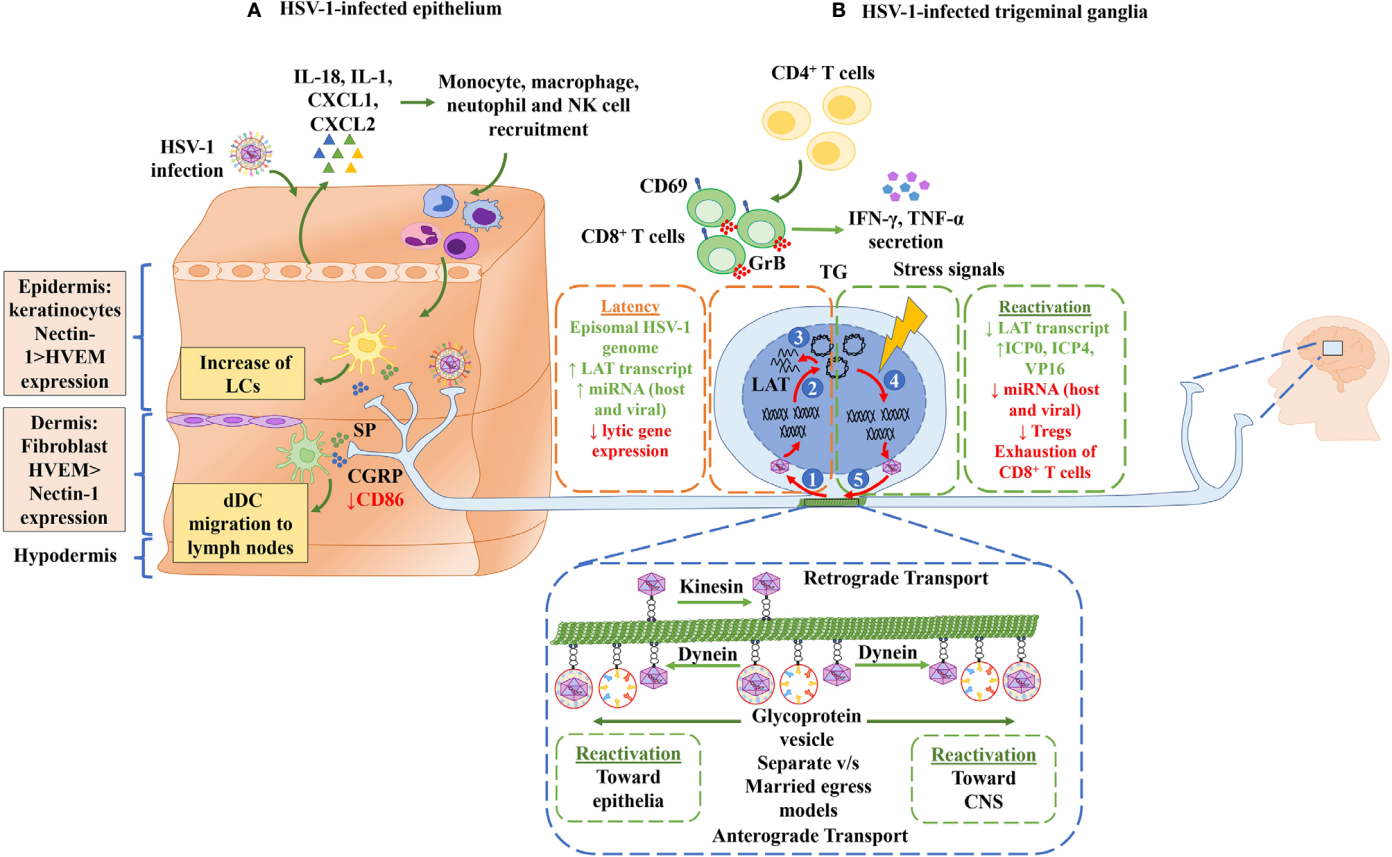
Neuroimmune crosstalk during HSV-1 infection. **(A)** HSV-1-infected epithelium. Keratinocytes in the epidermis are infected by HSV-1, which results in the secretion of cytokines (IL-18 and IL-1) and chemokines (CXCL1 and CXCL2) that allow the recruitment of immune cells, such as monocytes, macrophages, natural killer cells and neutrophils. Langerhans cells (LCs), which are also present in the epidermis, process viral antigens. Once LCs are activated, they migrate to the lymph nodes where they present viral antigens to T cells. In the dermis, fibroblasts and dermal DCs (dDCs) can also be infected by HSV-1. Moreover, the epidermis and dermis are innervated by the axons of sensory neurons. These axon terminals release substance P (SP) and calcitonin gene-related peptide (CGRP) neuropeptides that act as signaling molecules that activate LC and dDC migration to the lymph nodes. CGRP is recognized by CGRP receptors in dDCs, causing a decrease in the surface expression of the costimulatory molecule CD86 in dDCs. Finally, the innervation of nerve terminals in the epithelium allows HSV-1 to infect the neurons. **(B)** HSV-1-infected trigeminal ganglia. Once the axon terminals are infected by HSV-1 (1), the viral capsid associated migrates along microtubules by retrograde transport with kinesin proteins to the nuclear membrane. (2) In the nuclear membrane, the HSV-1 genome is injected into the nucleus. Once in the nucleus, the linear HSV-1 genome adopts an episomal configuration to establish latency in neurons. (3) In this episomal state, only LAT gene transcription occurs. LAT expression and the subsequent latent state are regulated by CD8^+^ T cells, which express CD69 and granzyme B (Grb). Moreover, CD4^+^ T cells secrete IFN-γ and TNF-α molecules that regulate the activation of CD8^+^ T cells. (4) Stress signals, such as UV light, psychological stress, menstruation, and fever, lead to an up-regulation of the expression of viral lytic genes and consequently, to the reactivation of HSV-1 infection. (5) HSV-1 components migrate through microtubules, by anterograde transport, to the epithelium or towards the central nervous system (CNS) through their association with the dynein protein in a “separate” or “married” egress model.

HSV-1 can enter the organism by interacting with skin epithelial cells, as the initial site of infection, by binding to heparan sulfate proteoglycans (HSPGs) on the cell surface thanks to the viral glycoprotein B (gB) and glycoprotein C (gC) (4). In turn, gB engagement allows glycoprotein D (gD) to bind one of its receptors, such as nectin-1 or nectin-2 in epithelial cells (5, 6), or the herpes virus entry mediator (HVEM), which is mainly expressed in immune cells (**Figure 1**) (7). Engagement of gD to one of its receptors will then induce the activation of the glycoprotein H/glycoprotein L (gH/gL) complex on the virion surface, which enables gB to act as the fusion protein allowing the viral and cellular membranes to combine leading to the subsequent entry of the viral capsid and tegument into the cytoplasm (**Figure 1**) (8). Once the viral DNA is injected into the nucleus, after the docking of the capsid to nuclear pores, viral gene expression occurs sequentially in a cascade-dependent manner: first immediate-early (IE, alpha) genes are transcribed, then early (E, beta) genes, and finally late (L, gamma) genes (**Figure 1**) (9, 10). These genes will allow the virus to escape immediate cellular antiviral responses, replicate the viral genome, and assemble new viral particles (11, 12). New copies of the viral DNA will be packaged into new capsids in the nucleus and traverse the nuclear membranes to access the cytoplasm, where they are complemented with additional tegument proteins and acquire an envelope with viral glycoproteins before exiting the cell in exocytic vesicles (**Figure 1**) (13). The new infectious viral particles released by skin epithelial cells can then gain access to type-C fibers of sensory neurons that innervate the skin and reach the cell body of neurons by retrograde axonal transport (14, 15). Alternatively, HSV-1 may infect neurons through close cell-cell contacts (16). Spread of the virus to sensory and autonomic nerve termini of neurons will create a reservoir of virus in the trigeminal ganglia (TG) or dorsal root ganglia (DRG), depending on the site of infection (17–19). Importantly, HSV-1 can enter a latency phase within neurons in which viral DNA remains as a circular episome in the nucleus and is characterized by the transcription of the latency-associated transcript (LAT), which encodes miRNAs that modulate viral gene expression (20–22). Nevertheless, the sporadic expression of lytic viral genes in neurons during latency, in the form of mRNAs has been reported by several groups, leading to the concept of HSV-1 molecular reactivation in these cells (23–25). In some cases, this is followed by protein synthesis but without the production of infectious viral particles (26, 27). However, under certain conditions, such as stress, HSV‐1 can reactivate within neurons and initiate the production of infectious particles that travel by anterograde axonal transport to the initial site of infection, causing secondary or recurrent lesions. Interestingly, HSV-1 anterograde migration occurs through either of two mechanisms: the “separate” model that proposes that the capsids containing the HSV-1 genomes and viral glycoproteins travel along microtubules separately and complete viral particles are formed at the terminal of axons (28), or the “married” egress model, which is proposed to be mediated by HSV-1 virions that travel as complete viral particles from the cell soma to nervous termini (**Figure 2**). In both cases, the newly synthesized viral particles can spread onto other cells, tissues and new hosts (2, 29). Noteworthy, HSV-1 reactivated from neurons of the TG is likely the primary source of the virus that causes herpetic encephalitis, although the virus may also access the brain after eye infections, which can lead to herpetic stromal keratitis (HSK) (30, 31).

Many studies have reported significant roles for immune components in the control of lytic infection by HSV-1 in epithelial cells and viral latency in neurons (2, 32–34). However, the intricate interactions occurring between immune cells and the nervous system at the skin and TG are somewhat poorly understood. Likewise, how the immune system is modulated by neuronal signals during HSV-1 infections remains to be fully determined. Here we review and discuss immune responses taking place in the skin and TG during HSV-1 infection, as well as neuro-immune interactions that are mediated mainly by soluble factors, such as neuropeptides, and that could regulate the immune response during the infection process.

## Immune Responses in the Skin During HSV-1 Infection

The skin is the largest organ in the body and includes three main layers: the hypodermis, the dermis, and the epidermis (35). Keratinocytes are the most superficial and primary cell type within the epidermis and protect the body against microbial infections by producing keratin and secreting defensins (36). Keratinocytes are arranged in layers according to their differentiation stage, with new cells in the deepest layer (stratum basal) and the oldest cells in the upper layer (stratum corneum), which contains dead cells called corneocytes (37). Above the stratum basal there is a network of a dendritic cell (DC) subtype named Langerhans cells (LCs), which act as sentinels at this site, sensing microbes and foreign antigens (38). Importantly, these types of immune cells have been identified as key players in the defense against HSV-1 infections (39). Upon HSV-1 skin infection, an increase in the number of LCs is observed in this tissue, as evidenced in the footpads of mice and the mucosal epidermis of animals infected with this virus (40). In the underlying dermis, fibroblasts are the primary resident cell type and are also susceptible to HSV-1 infection (41). Here, dermal DCs (dDCs) are also present and have been characterized as key players in viral antigen presentation. These cells can also migrate to the draining lymph nodes to activate T cell responses (38, 42). Noteworthy, type-C nervous fibers of sensory neurons from the TG are also present in the skin, innervating the epidermis and dermis and are likely the main portal of entry for HSV-1 from the periphery into the central nervous system (CNS) (**Figure 2A**) (15).

Seemingly, HSV-1 can infect epidermal keratinocytes once the virus penetrates the stratum corneum, either through nectin-1 or HVEM, as both receptors are expressed in these cells (43). The virus is then propagated either through viral particles released by infected cells or through cell-to-cell contact, and consequently can reach the dermis where fibroblasts may also be infected (41). However, some differences are observed regarding the expression of HSV-1 receptors in these cells: while nectin-1 is less expressed in fibroblasts than in keratinocytes, HVEM is present in almost all fibroblasts, but only expressed on a limited number of keratinocytes in the epidermis (**Figure 2A**) (7). Nevertheless, nectin-1 has been reported to be the central receptor for HSV-1 entry into both cell types in murine skin, and thus, the role for HVEM at this site is somewhat unclear at this time. Actually, HVEM may relate to other effects over the immune response that are mediated by this virus (43). Interestingly, changes in HVEM expression following ocular infection were reported to modulate HSV-1 spread and inflammation in the cornea of HSV-1-infected mice (44). Alternatively, studies in mice have shown that HSV-1 also enters into epidermal cells via endocytic vesicles, and another study using human keratinocytes supports endocytic uptake during HSV-1 infection (45).

The infection of epithelial cells has been reported to lead to the production of several cytokines, including interferons (IFNs), interleukins (IL) IL-18, IL-1, and chemokines such as KC (CXCL1) and MIP-2 (CXCL2) (46–48). In turn, this inflammatory environment mediates the recruitment of innate immune cells, which could help counteract viral propagation (**Figure 2A**) (47, 49, 50). Indeed, a study reported that keratinocytes release IL-1α after HSV-1 skin infection leading to the early recruitment of leucocytes, which was associated with better outcomes (47). Consistently, IL-1R1-KO mice displayed an elevated mortality rate associated with viral dissemination, together with reduced neutrophil and monocyte/macrophage recruitment at the site of HSV-1 infection (47). Previous studies had also investigated the role of leukocyte infiltration during HSV-1 infection in the skin of mice and reported that Gr-1^+^ cells, other than Ly6G^+^ neutrophils, could inhibit the replication of HSV-1 (49). Although macrophages and natural killer (NK) cells infiltrate epidermal layers of the skin to a lesser extent than neutrophils, these cells have been reported to be key inflammatory mediators for controlling HSV-1 skin infections (50, 51). Interestingly, an increase in mortality rate and in viral loads in the brain, liver and spleen was observed in mice depleted of NK cell activity (50). Moreover, NK cells have been reported to act as non-cytotoxic helpers in the absence of CD4^+^ helper T cells, enhancing CD8^+^ T cell responses by releasing interferon (IFN)-gamma (IFN-γ) and/or IL-15 and IL-12 that in turn could contribute to effective adaptive immune responses against viral infections (52). It has been suggested that a reciprocal crosstalk between epithelial cells and leucocytes may occur as a cooperative process for promoting wound healing and enhance cellular antiviral responses (53).

Notably, neuronal function can also be regulated by cytokines produced during HSV-1 skin infections, and reciprocally, cytokines and neuropeptides released by neurons can affect the immune responses against HSV-1 (54, 55). In this regard, a recent study reported that pseudorabies virus (PRV), an *alphaherpesvirus* closely related to HSV-1, induced high levels of the granulocyte colony-stimulating factor (G-CSF) and IL-6 in the peripheral nervous system (PNS) and CNS tissue at early time-points after footpad inoculation with the virus, which occurred independent of viral replication in neurons, and resulted in systemic inflammation (56). The authors reported that PRV replication in the peripheral epithelial tissue was sufficient to activate DRG neurons to produce cytokines, leading to a lethal and severe neuropathy in mice. Furthermore, it was proposed that G-CSF and IL-6 could be transmitted to the vagus nerve leading to dysfunction of organs (56). In line with this notion, our laboratory recently reported that asymptomatic HSV-1 infection, after intranasal inoculation of mice with wild-type or a mutant virus deficient in neuronal replication, increases the expression of proinflammatory cytokines in the brain and spinal cord, and induces long-term alterations to the blood-brain barrier. When induced to undergo experimental autoimmune encephalomyelitis (EAE), which parallels aspects of multiple sclerosis in humans, these mice displayed increased disease severity (57). Furthermore, the animals inoculated with the mutant virus displayed an earlier disease onset and increased neuroinflammation (57). These studies suggest a direct relationship between herpesvirus infections with neuroinflammation and neurodegenerative diseases, and may explain some of the detrimental effects caused by viral infections over the CNS. Thus, CNS damage by herpesvirus infection may be related to a crosstalk between the immune system and the nervous system, rather than by direct effects of HSV-1 over cells in this tissue.

Notably, recent studies have reported that the intercommunication between the immune and nervous systems, as well as with epithelial cells in the skin, could elicit distinct effects over the control of lytic HSV-1 infection, as well as the establishment of latency by this virus (15, 54, 58, 59). However, it remains somewhat unclear how the signals elicited by sensory neurons, skin cells and immune cells are integrated within the skin to modulate immune cell function and epithelial cell responses during primary or recurrent HSV-1 infections.

## Immune Responses in HSV-1-Infected TG

### Establishment and Maintenance of HSV-1 Latency

Although the exact mechanism by which HSV-1 establishes lifelong infections in neurons of the TG in a latent state has many unknowns, there is evidence that the immune system has a substantial role in this viral process (32, 60, 61). Chronic immune cell infiltration has been described to accompany HSV-1 latency in TGs, with human TG sections displaying HSV-1 protein expression and a high amount of infiltrating T cells (62). After primary skin infection, virus spread is likely controlled by the innate immune response at this site, which limits the replication of the virus until an adaptive immune response is established, which will contribute to its clearance. Likely, these immune components at the skin will pressure the virus towards the initiation of viral latency, which requires neuron infection and retrograde transport of viral components to the nucleus of these cells (61). Indeed, CD8^+^ T cells have been reported to have an important role in the induction of HSV-1 latency in neurons at the TG, as the depletion of these cells has been shown to significantly prevent this viral process (17, 62, 63). Although other immune cell types, such as CD4^+^ T cells and neutrophils at this site are less frequently observed than CD8^+^ T cells, these cells may nevertheless play a relevant role in HSV-1 latency (64). For instance, CD4^+^ T cells have been reported to enhance CD8^+^ T cell responses to HSV-1, as CD4^+^ T cell-depletion in C57BL/6 mice impaired the production of IFN-γ and TNF-α by CD8^+^ T cells (**Figure 2B**) (65), suggesting that CD4^+^ T cells are essential for the generation of fully functional CD8^+^ T cells during primary HSV-1 infection. On the other hand, DCs may also play a prominent role in the establishment of viral latency by increasing the amount of LAT in the TGs of mice (33, 66, 67). A study using a transgenic mouse that expresses a CD11c-diphtheria toxin receptor, which allows the depletion of DCs after diphtheria toxin injection, showed that the depletion of these cells leads to reduced LAT expression in mouse TGs 30 days post-ocular infection (33). Conversely, an increase in the numbers of DCs, thanks to the administration FMS-like tyrosine kinase 3 ligand (Flt3L) DNA in mice, produced an increase in the amount of virus latently-infecting the neurons in the TGs of mice (66). Moreover, adoptive transfer experiments with DCs expanded *ex vivo* with Flt3L, or GM-CSF, and then transferred into recipient mice displayed increased the LAT expression in the TGs, a process associated with lymphoid-related DCs (CD11c^+^CD8α^+^) (66). These reports suggest a close interplay between DCs and neurons, and nearby interactions that seemingly favor neuron infection with HSV-1 during skin infection with this virus. These findings suggest that DCs may be used as Trojan horses by HSV-1 to access neurons, which are the permanent reservoir for this virus in the host (68).

Immune soluble factors have also been shown to modulate HSV-1 latency in neurons. Indeed, transforming growth factor-β (TGF-β), a negative regulator of the innate and adaptative immune response, has been described to play a role in enhancing HSV-1 latency in the trigeminal ganglia of infected mice. A study that blocked TGF-β signaling in innate cells (CD11c^+^ cells) or T cells (CD4^+^ and CD8^+^ T cells) resulted in a reduction of latency in the TGs of ocularly-infected mice, which was inferred by a decrease in the amount of LAT expression in neurons (69). Moreover, TGF-β signaling blockade in T cells decreased cell-mediated lysis in the infected corneas and reduced leukocyte infiltration into TGs during primary HSV-1 infection in mice (70).

### Regulation of HSV-1 Reactivation

The shift between latent infection and reactivation of the HSV-1 lytic cycle likely occurs after virus and cell components sense either directly or indirectly neuronal signals induced by stimuli, such as exposure to UV light, psychological stress, menstruation, or fever, among others, that lead to the up-regulation of the expression of viral lytic genes (71).

Although the reactivation process of HSV-1 has been very studied, it is yet to be fully understood. miRNAs have been reported to be important regulators of gene expression during latency, as summarized in **Table 1**. A neuron-specific miRNA, namely miR-138 has been reported to regulate the expression of ICP0 in latently-infected cells (72). This viral protein enhances viral reactivation and thus, a down-regulation of miR-138 may promote viral exit from the latency state. On the other hand, LAT acts as a miRNA precursor that encodes four different miRNAs that regulate the expression of viral lytic genes (22). Three of these miRNAs are antisense for HSV-1 mRNAs and specifically target the mRNAs related to ICP0 (miR-H2) and ICP34.5 (miR-H3 and miR-H4) (22). Notably, miR-H2 was shown to inhibit ICP0 protein expression (22). An HSV-1 mutant with disrupted expression of miR-H2 was described to display increased levels of ICP0 expression and showed an increased rate of reactivation in mouse TG explants used as an *in vitro* model for virus reactivation (74). Furthermore, several studies have reported that ICP0 plays a crucial role in HSV-1 reactivation. A study performed with multiple viruses encoding ICP0 mutations reported that this protein is needed during reactivation in latently-infected ganglia to enhance the number of viral genomes in these tissues and the transactivating activity of ICP0 (77). Another study found that not only ICP0 expression was sufficient for HSV-1 reactivation in latently-infected TG cell cultures, but also the expression of ICP4 or VP16 was enough to induce this process (78). Interestingly, the HSV-1 miRNA miR-H6 has been shown to be partially complementary to the mRNA of ICP4, leading to decreased expression of the corresponding viral protein which is a transcription factor that is essential for the expression of most HSV-1 genes during the lytic infection cycle and for the induction of HSV-1 reactivation in primary cultures of latently-infected TG (22, 78). Moreover, explants of latently-infected DRG with a virus lacking miR-H1 and miR-H6 was reported to display lesser viral reactivation when compared to the WT virus (73). Moreover, rabbit ocular infection with this mutant virus displayed impaired epinephrine-induced reactivation (73). Deletion of these miRNAs also promoted the accumulation of LAT (73). On the other hand, the effects of the viral miRNAs miR-H3 and miR-H4 have not been assessed in the context of viral reactivation and thus, their roles in this process remain unknown (22, 76). Moreover, a study reported that miR-H8, which is antisense to the gene of ICP0, had no detectable effect on the loads of viral genome during latency, or viral reactivation (79).

**Table 1:** Summary of miRNA modulation of HSV-1 reactivation.

miRNA (cellular or viral)	miRNA effect	Regulation of HSV-1 reactivation	Reference
**miR-138 (cellular)**	Inhibits ICP0 expression.	Down-regulation of miR-138 promotes reactivation.	(72)
**miR-H1 (viral)**	Targets the host intrinsic effector, ATRX, component of ND10-bodies.	A deletion of the seed sequences of miR-H1 reduces reactivation.	(73)
**miR-H2 (viral)**	Inhibits ICP0 expression.	A mutant with miRNA-H2 disrupted showed increased rate of reactivation.	(74, 75)
**miR-H3 (viral)**	Antisense to the ICP34.5 gene and likely inhibits its expression.	Unknown	(22)
**miR-H4 (viral)**	Inhibits ICP34.5 protein expression.	Unknown	(22, 76)
**miR-H6 (viral)**	Inhibits ICP4 protein expression.	A deletion of the seed sequences of miR-H6 reduces reactivation.	(22, 73)
**miR-H8 (viral)**	Antisense to the first intron of ICP0	Dispensable for viral reactivation *in vivo*.	(22, 73)

There is also evidence that *de novo* expression of the viral protein VP16 is necessary for HSV-1 exit from latency in neurons. Interestingly, it has been shown that this protein is not efficiently transported to the nucleus of neurons, where it carries out its main functions as a transactivator, and therefore is not present during latency. Also, VP16 has been described to be only expressed when repressors encoded by the LAT locus in lytic genes are overcome (80). Thus, if sufficient amounts of VP16 are expressed, this factor may coordinate the expression of IE genes (i.e. ICP4 and ICP0). Interestingly, repression of VP16, which is encoded by U_L_48, a late gene, can be suppressed thanks to a positive feedback loop upon its expression. Consequently, viral reactivation may occur in neurons, where infectious virus is produced and expanded within the ganglia, with viral particles then traveling back to the original site of infection (80).

Interestingly, some studies have reported that the cell coactivator termed host cell factor-1 (HCF-1), which is found in the cytoplasm of sensory neurons, is transported to the nucleus of these cells in response to HSV-1 reactivation stimuli, providing some potential clues regarding the molecular reactivation of HSV-1 from neurons (81). After HSV-1 reactivation stimuli, such as TG explantation in tissue culture dishes, HCF-1 has been detected in the promoter region of viral IE genes, suggesting that HCF-1-dependent complexes might contribute to viral reactivation (82). The role of c-Jun N-terminal kinase (JNK), which is related to the signaling of several stress responses has also been studied in HSV-1 reactivation (83). The activation of this kinase induces a histone methyl/phospho-switch that acts over HSV-1 lytic promoters, even under repressive conditions through lysine methylation of histones, thus allowing viral gene expression under these conditions (83). Remarkably, an *in vivo* study with mice showed that there is a positive correlation between the number of latently-infected neurons in the TG and the likelihood of viral reactivation; therefore, the number of latent genome copies may have an impact on the efficiency of viral reactivation *in vivo* (84).

Interestingly, there are also numerous studies relating the immune system to HSV-1 exit from latency. For instance, some studies suggest that the productive reactivation of HSV-1 from neurons is inhibited by CD8^+^ T cells that co-localize nearby latently-infected cells in the TG, both in mouse and human tissues (19, 85). Furthermore, it is known that these T cells recognize HSV-1 epitopes, specifically the gB_498-505_ epitope in mice, which is expressed during episodes of viral molecular reactivation. CD8^+^ T cells expressing CD69 and granzyme B, which are indicative of cell activation after recent antigen exposure, are found adjacent to neurons infected with HSV-1 (63, 86). The importance of CD8^+^ T cells has been further illustrated in a study in which the supplementation of infected TG cultures with exogenous CD8^+^ T cells delayed the reactivation of HSV-1, and on the contrary, when these cultures were treated with an anti-CD8 antibody, which blocks the function of these cells, they displayed an increase in viral reactivation (63).

Furthermore, it has been reported that CD8^+^ T cell lytic granules are required for blocking the HSV-1 replication cycle and that granzyme B (GrB) cleaves the viral protein ICP4, which is needed for the transcription of early and late viral genes (87). Moreover, in the TG of mice that are deficient in lytic granule components, namely perforin or GrB, an increase in viral genome copies was observed (87). Another study described an important role for the viral protein ICP22, showing that ocular infection of mice with a virus lacking this protein displayed lower viral titers than animals infected with the wild-type virus (88). Also, latency and reactivation were reduced in mice infected with the mutant virus (88).

Interestingly, PD-L1 expression, a T-cell marker that binds to co-stimulatory molecules such as CD80, was also downregulated in latently-infected TG when infected with an ICP22 null virus (88). Additionally, a study with mice that do not express IFN-γ or the IFN-γ receptor showed no significant differences in viral titers, or the time required to establish latency in the TG when compared to IFN-γ-competent mice (89). However, upon hyperthermic stress, HSV-1 antigens were found to be expressed in multiple neurons in IFN-γ-deficient mice and only in a single neuron in the control mice, and was followed by a higher frequency of viral reactivation in the IFN-γ-incompetent mice (89). This finding suggests that IFN-γ may not control the viral reactivation process per se, but might have a virus-limiting effect immediately after its reactivation (89). There is also evidence that a reduction in CD8^+^ T cells induces HSV-1 reactivation in stressed mice through a regulatory effect that is mediated by Treg cells, which in turn allows the expression of viral genes, such as ICP0 and ICP4 (90).

It is important to note that more studies focused on the regulation of the immune response by neuronal signals are needed, namely on the characteristics of resident CD8^+^ T cells and other immune cells that surround the infected neurons in the TG. This knowledge could help better understanding the dynamics of the viral reactivation processes and the molecular and cellular pathways that govern HSV-1 exit from latency.

## Neuronal Signals Regulating the Immune System and HSV-1 Infection

Neuronal interactions occur mainly through neurotransmitters, but these cells can also transmit signals to other cell types through neuropeptides that regulate distinct physiological functions, and may also participate in pathological conditions (91, 92). During HSV-1 infection, such molecules may play critical roles in lytic infection and the establishment and maintenance of viral latency in neurons by modulating antiviral immune components (93). Some neuropeptides expressed by sensory neurons of the TG are tachykinins, calcitonin gene-related peptide (CGRP), vasoactive intestinal peptide (VIP), somatostatin (SST), galanin, and neuropeptide Y, which we review and discuss below and are summarized in **Table 2** (94, 95).

**Table 2:** Summary of neuropeptide effects on immune cells and HSV-1-infected cells.

NEUROPEPTIDE	EFFECTS ON IMMUNE CELLS	EFFECTS ON HSV-1-INFECTED CELLS
**Substance P** **(SP)**	Memory CD4^+^ T cells: Induction of IL-17A and IFN-γ expression.	Corneas with herpetic stromal keratitis (HSK): Induction of IL-6, IFN-γ, CCL3, CXCL2 expression.
	Peripheral blood human T cells: Induction of IL-2 expression.	Macrophages: Induction of IL-1β expression.
**Calcitonin gene related peptide (CGRP)**	CD4^+^ T cells: Decreases IL-2 and IFN-γ expression.	Primary neuronal cultures of TG: HSV-1 LAT expression induces CGRP expression.
	Dendritic cells: Elicits an immature state, induces DC migration.	Trigeminal ganglia sensory neurons: HSV-1 latency reduces CGRP expression.
**Somatostatin** **(SST or SS)**	Activated monocytes: Inhibition of TNF-α and IL-1β expression.	Unknown
	B cells: Inhibition of IgE secretion in tonsils.	
	Intestinal mucosa T cells: Induction of IL-12 expression.	
	Mouse T cell line: Induction of IL-4 and IL-10 expression.	
	Peripheral blood T cells: Decrease of IL-12 expression.	
	Splenocytes: Decrease of IFN-γ expression.	
**Vasoactive Intestinal Peptide (VIP)**	LPS-stimulated Macrophages: Induction of IL-6 and IL-10, which produces CD4^+^ T cell polarization from a Th1 profile towards a Th2 profile.	Unknown
	Macrophages: Inhibition of TNF-α, IL-2, IL-6 and IL-12 expression, and antigen presentation.	
	Murine T cells: Decrease of IL-2, IL-4 and IFN-γ expression.	
**Galanin**	Human macrophages: Induction of IL-10, IL-1Ra, TGF-β, CCL3 and CXCL8 expression.	Trigeminal ganglia sensory neurons: HSV-1 infection induces an increase in galanin expression after corneal infection.
	Neutrophils: Induction of IL-8 expression.	
	NK cells: Induction of IL-18 and IFN-γ expression.	
	Non-activated monocytes: Induction of IL-10, IL-18, IL-1 β, TNF-α, CCL3 and CXCL8. expression	
**Neuropeptide Y (NPY)**	B cells: Decreases Fas (CD95) expression.	Unknown
	Dendritic cells: Decreases CD80 and CD86 co-stimulatory molecules expression.	
	Immature Dendritic cells: Induction of IL-6 and IL-10 expression.	
	LPS-activated macrophage: Decrease of TNF-α and Induction of TGF-β1 expression.	
	Macrophages: Decrease of IL-6, TNF-α and nitric oxide synthase 2 (NOS2) expression.	

### Substance P

Substance P (SP) belongs to the family of tachykinins, which includes neuropeptides expressed by neuronal and non-neuronal cells (96, 97). Interestingly, T lymphocytes and monocytes bear specific receptors for this neuropeptide, mainly the neurokinin 1 (NK-1) receptor (96, 98). Noteworthy, SP has been reported to modulate the phenotype and function of these cells (99, 100). For example, peripheral blood human T cells stimulated with PHA plus PMA (T cell activators) in the presence of SP increased the expression of IL-2, a cytokine indicative of T cell activation (99, 101). Moreover, SP also increased the production of IL-17A and IFN-γ by human memory CD4^+^ T cells, which favored their polarization towards a Th17/Th1 profile that was dependent of monocyte-derived proinflammatory cytokines (102). In addition, there is growing evidence involving SP, as well as its receptors, in the pathophysiology of several inflammatory disorders and infections due to their capacity to regulate immune responses (103–105). Regarding HSV-1 infection, an *in vitro* assay reported increased production of IL-1β in HSV-1-infected mouse peritoneal macrophages in the presence of very low concentrations of SP (10^-9^ M) (106). Also, in a mouse model of HSK, SP levels were significantly higher in the corneas of mice with severe HSK, as compared to animals with mild lesions. Corneas with higher levels of SP were associated with more significant inflammation of this tissue and had increased amounts of IL-6 and IFN-γ, as well as the chemokines CCL3 and CXCL2 (107). Treatment with spantide I, an antagonist of the NK-1 receptor, induced a significant reduction in corneal opacity and angiogenesis, as well as in the expression of IL-6 and CCL3 in the infected corneas (107). Surprisingly, a later study that evaluated the role of the NK-1 receptor on the severity of herpetic keratitis reported that mice that lack a functional NK1R receptor (NK1R^-/-^) display an earlier development of severe HSK, with increased amounts of CD4^+^ T cells and neutrophils infiltrating the corneas (108). The implications of SP in HSV-1 ocular infection are also supported in another study in which chemical sympathectomy was induced in mice by administering 6-hydroxydopamine hydrobromide (6OHDA) before viral infection mediated by corneal scarification. Because 6OHDA treatment induces changes in the innervation of the corneas, which could alter the host's responses to pathogens, the authors hypothesized that 6OHDA could also modulate the course of HSV-1 infection. Interestingly, they found a significant rise in SP levels and a decrease in the expression of IFN-γ in TGs of 6OHDA-treatead mice, which was partially reduced when mice were administered an NK1R antagonist (109). While the use of NK1R antagonists is being tested in several clinical trials for the treatment of other pathologies (110, 111), further studies are needed to fully elucidate the role of SP and its receptor in HSV-1 eye infections and their potential therapeutic uses. Given the effects reported so far for SP in HSV-1 infection in experimental laboratory settings, we foresee that such drugs could play a favorable role in decreasing HSV-1-mediated inflammation in the eye.

On the other hand, it has been reported that HSV-1 latency may alter the expression of some neuronal genes in the trigeminal sensory neurons (112). Consistent with this notion, a study using cultures of TG neurons transfected with an expression vector encoding the 2.0 kb-long LAT transcript was related to a higher percentage of SP-immunoreactive neurons, as compared to the control-transfected neurons. This result suggests that HSV-1 LAT can modulate, either directly or indirectly, the expression of neuropeptides in trigeminal sensory neurons (113). However, it remains to be determined if this effect, over latently-infected neurons, has a role in maintaining latency or over the symptoms associated with postherpetic neuralgia, which is frequently reported in individuals with recurrent HSV-1 infections (114).

### Calcitonin Gene-Related Peptide

Calcitonin gene-related peptide (CGRP) is another neuropeptide that is highly expressed within TG and co-localizes with SP in most neurons (94, 115). The CGRP receptor consists of three subunits: receptor activity-modifying protein 1 (RAMP-1), calcitonin-like receptor (CLR) and receptor component protein (RCP), which is widely expressed in the cardiovascular system where it is supposed to exert protective functions, as well as in different areas of the nervous system and has been related to migraine (116). This neuropeptide is also recognized for its ability to modulate immune responses and has been implicated in neurogenic inflammation and pain-related neuropathic pathways (116).

Contrary to SP, CGRP has been shown to decrease the production of IL-2 and IFN-γ in CD4^+^ T cells, and thus more likely induces a Th2 polarization profile in these cells (117–119). Interestingly, CGRP has also been described to inhibit IL-13 production in type-2 innate lymphoid cells (ILC2s), as well as their proliferation and to induce a regulatory gene expression profile (120). *In vivo* studies showed that CGRP limits ILC2-dependent airway inflammation and that the deletion of one of the CGRP receptor chains, namely the *Ramp1* gene, stimulates Th2 immune responses (120). In contrast, CGRP has been shown to enhance IL-1β, IL-6 and TNF-α secretion in a concentration-dependent manner in lymphocyte-enriched mononuclear cells derived from human peripheral blood (121), and have a synergic effect over TNF-α secretion when combined with SP (121). CGRP has also shown to modulate cytokine production in the context of bacterial infections. In response to methicillin-resistant *Staphylococcus aureus* infection, treatment with CGRP inhibited TNF-α and CXCL1 by lung cells and the levels of this neuropeptide significantly increased after infection (120, 122). During *Salmonella enterica* serovar Typhimurium TRPV1^+^ nociceptors were reported to regulate bacteria loads via CGRP (123). Furthermore, nociceptors were reported to release CGRP and inhibit the recruitment of neutrophils to the infection site and opsonophagocytic killing of *Streptococcus pyogenes* bacteria, evidencing that this neuropeptide can modulate immune function and host response to pathogens (124).

Notably, HSV-1 latency in neurons seems to affect the expression of CGRP by sensory neurons in the TG, although the role of this neuropeptide during this viral process remains controversial. Noteworthy, the expression of CGRP was found to be reduced in primary neuronal cultures of TG obtained from rats transfected with a plasmid expressing LAT (125), which differs from the results obtained in another study using human TG in which CGRP was more frequent in LAT-positive neurons than LAT-negative neurons, as detected in immunofluorescence assays (126).

On the other hand, a study found that CGRP displays the capacity to stimulate the migration of T cells within a collagen matrix, which suggests that this neuropeptide may enhance the ability of T cells to migrate into inflamed tissues, by passing through endothelial cells (127). Another study reported that CGRP can act as a chemoattractant for DCs and elicit an immature state in these cells. Thus, CGPR could be related to the migration of these immune cells toward sites of inflammation produced by HSV-1 (128). Moreover, it has also been reported that CGRP induces IL-23 production by dDCs, which was shown to further lead to IL-17 release by γδ-T cells that mediate neutrophil recruitment after skin infections (129). Interestingly, several studies have shown that DC migration is influenced by sympathetic and sensory neural signals in different tissues, including the skin, as assessed by confocal microscopy (130, 131). Previous studies using this technique showed that epidermal LCs were located nearby CGRP^+^ type-C fibers in the epidermis, suggesting interactions between nervous system cell and immune cells in the skin (132). Another study, reported that CGRP binding to its receptor, on the surface of human immature and mature DCs, inhibits the expression of co-stimulatory molecules in these cells such as CD86, suggesting that CGRP may downplay T cell stimulation by DCs (133). Because HSV-1 infection has been associated with the downregulation of molecules that modulate the interaction of DCs with other immune cells, such as CD1a, CD40, CD54 (ICAM-1), CD80 and CD86, it is possible that the neuropeptide CGRP may further exert inhibitory effects over DCs after infection with HSV-1 (134, 135). However, CGRP was reported to inhibit HSV-1 replication in the skin of the footpads of mice after infection, which was reverted after the treatment with an antagonist of CGRP. Indeed, this treatment recapitulated similar viral loads as compared to control mice injected with the virus alone (136). Thus, studying more closely the relationship between DCs and neuropeptides released by nerve endings of sensory neurons from the TG may help draw a better picture of the neuroimmune regulation occurring during HSV-1 infection and help designing new and more effective drugs, or vaccines prospects against this virus, because these cells play important roles at mounting protective antiviral adaptive immune responses to HSV-1 (137, 138).

### Vasoactive Intestinal Peptide

Vasoactive Intestinal Peptide (VIP) has been identified as an important modulator of the immune system that is moderately expressed by neurons in the TG (94, 139). Interestingly, receptors for VIP, namely VPAC1 and VPAC2, have been reported to be expressed on a variety of immune cells (139, 140). However, the effects of VIP over immune cells have been mostly studied with macrophages. Importantly, VIP was found to significantly inhibit the production of cytokines, such as TNF-α, IL-12, IL-6, and IL-2, and antigen presentation by macrophages (140–142). The anti-inflammatory effects elicited by VIP over other immune cells relate to decreased expression of IL-4, IL-2, and IFN-γ in stimulated murine T lymphocytes (141). Promotion of CD4^+^ T cell polarization towards a Th2 phenotype from a Th1 phenotype has also been reported by VIP, which was mediated by an increase in the expression of IL-10 and IL-6 in LPS-stimulated macrophages (143). In contrast, a proinflammatory role was found for VIP in DCs, as VIP induced the maturation of human DCs by synergizing with the proinflammatory cytokine TNF-α (144). Furthermore, findings on the role of VIP in the differentiation of CD4^+^ T cells to a Th17 phenotype are contradictory. In experimental models of autoimmune diseases, such as type-I diabetes, collagen-induced arthritis and EAE, the administration of VIP inhibited Th17 differentiation, as evidenced by a reduced expression of IL-17, the transcription factor RORγt and IL-22, which delayed disease onset (145, 146). However, increased differentiation of murine CD4^+^ T cells toward a Th17 profile has been observed in an *in vitro* assays using Langerhans cells as antigen presenting cells in the presence of TGF-β and VIP, as well as with naïve human T cells during their differentiation (147–149).

Noteworthy, at present there are no studies reporting a role for VIP over HSV-1 infections or vice-versa. Nevertheless, it seems paramount not only to evaluate the impact of VIP during the course of infection, but also over the function of different immune cells involved in HSV-1 infection for determining whether VIP can exert a proinflammatory or anti-inflammatory effect in the context of the host immune response against infection with this virus, both during the lytic and latent cycle of the HSV-1.

### Somatostatin

Somatostatin (SST or SS) is a hormone and neuropeptide that predominantly inhibits immune system functions (150). Binding sites for this neuropeptide have been found in several immune cells, including murine B and T cells in the spleen and in humans in the lymph nodes, thymus, and the spleen (151). Interestingly, in normal human T cells, SST has been reported to stimulate or inhibit IL-2 secretion depending on the cells' origin. T cell stimulation was seen in lymphocytes purified from the intestinal mucosa (152), while inhibition was reported for lymphocytes purified from peripheral blood (153). Additionally, it has been shown that SST stimulates IL-4 and IL-10 production by a mouse T cell line (154). However, IFN-γ secretion was found to be inhibited by SST in granuloma T cells stimulated with a mitogen (155). On the other hand, SST was found to inhibit the secretion of immunoglobulins (Ig), such as IgE in the tonsils (156), as well as IgG, IgA, and to a lesser extent IgM in B cells obtained from peripheral blood and the intestinal mucosa (152). Furthermore, SST has been reported to have antitumor and anti-inflammatory effects and to inhibit angiogenesis in endothelial cells and was shown to lead to the inhibition of growth factor-stimulated and mitogen-activated protein kinase (MAPK) pathways, as well as the activity of endothelial nitric oxide synthase (NOS) (157).

Somatostatin has also been reported to have an inhibitory effect on the secretion of inflammatory cytokines, such as TNF-α and IL-1β by activated monocytes purified from human peripheral blood (158). Interestingly, SST is also involved in the inhibition of human NK cell activity (159), and inhibits human neutrophil chemotaxis (158). Because these innate immune cells are involved in the control of primary HSV-1 infections, as described above, SST could induce an indirect negative effect over the course of infection and facilitate viral spread, which remains to be evaluated.

### Galanin

Galanin is a widely distributed peptide that is found in the CNS and PNS, as well as in non-neural tissues (160). Galanin is suggested to be a nociceptive modulator of inflammatory pain (161, 162). Treatment with exogenous galanin has been reported to increase the expression of anti-inflammatory cytokines such as IL-10, IL-1Rα, and TGF-β, and the chemokines CCL3 and CXCL8 by human macrophages (163). This effect was also seen in non-activated monocytes, where the treatment with galanin increased the expression of IL‐10, IL‐18, IL‐1β TNF‐α, CXCL8 and CCL3. However, this effect was not seen for macrophages (163). Furthermore, this neuropeptide has also been reported to affect immune responses mediated by activated monocytes, by regulating their cytokine expression (164), and neutrophils by acting as a modulator of their activation (165). Interestingly, galanin improved the response of NK cells to IL-18 and modulated the production of IFN-γ by cytokine-producing NK cells upon IL-18 and IL-12 stimulation. However, galanin did not affect NK cell cytotoxicity, nor did it affect the dynamic mass redistribution of this cell type (166). Regarding galanin's effect over neutrophils, it has been reported that this neuropeptide increases the response of polymorphonuclear neutrophils to IL-8, but that this treatment was not able to induce CD11b integrin surface expression, which is used to evaluate receptor-ligand interactions (165). It is important to highlight that HSV-1 induces changes in galanin expression in TG neurons. Six days post-corneal viral inoculation, an increase in galanin expression could be observed until day 10 post-infection, when this factor began to decrease until reaching normal levels on day 21 after infection (167). However, although galanin is induced by HSV-1 in this tissue, the effects of this neuropeptide on the course of disease, as well as on the establishment and maintenance of latency in the TG, have not been evaluated. Thus, future studies assessing this factor on HSK and other HSV-1-related diseased could help to identify new pharmacological targets.

### Neuropeptide Y

Neuropeptide Y (NPY) has been reported to participate in numerous biological processes, such as food intake and energy metabolism, as well as inflammatory processes. This peptide is widely distributed in the nervous system, and it is suggested to be a transmitter between the immune and nervous systems, being mainly produced and released by immune cells (168). It has been reported that under inflammation conditions, the secretion of NPY by structural, neurogenic, and immune cells is elevated, leading to immune cell function regulation (169, 170). Interestingly, NPY preserves human T and B cells from undergoing apoptosis, and stimulates their proliferation. NPY serum levels negatively correlate with the expression of Fas/Fas ligand in T cytotoxic and T helper cells, and also negatively correlates with the expression of Fas in B cells (171, 172). Additionally, a negative correlation exists between NPY plasma levels and NK cell activity (173). Interestingly, the effects of NPY over NK cells appear to be somewhat dose-dependent, as large doses of this neuropeptide enhanced the mobilization of activated NK cells towards the blood. In contrast, low doses reduced the number of NK cells in the blood (174).

Regarding DCs, NPY was shown to inhibit the expression of co-stimulatory molecules on the surface of these cells, such as CD80 and CD86 (175). Additionally, this neuropeptide conferred a Th2-polarizing profile to DCs, due to the up‐regulation and production of anti-inflammatory cytokines, namely IL-6 and IL-10 (176). At physiological concentrations, NPY has been described to increase the recruitment of monocytes and macrophages, as well as their adherence capacity to tissues (177–180). However, the pro-adhesive effects of this neuropeptide were not seen in aged mice, nor rats previously exposed to stress. Additionally, it has been shown that NPY affects cytokine release by macrophages and monocytes. Indeed, after *in vitro* stimulation with concanavalin A (a non-specific T cell activator), this neuropeptide promoted IL-1β secretion by mouse peritoneal macrophages (181, 182). A similar effect was reported for macrophages derived from human peripheral blood (183). Furthermore, following stimulation with LPS, NPY decreased macrophage production of TNF-α and increased the production of TGF-β1 (181, 184). Noteworthy, NPY is produced by macrophages that are present in the adipose tissue, and the injection of this neuropeptide into lean mice reduced the quantity of M1-like proinflammatory macrophages in this tissue (185).

To our knowledge, at present there are no studies assessing the potential effects of this neuropeptide during HSV-1 infection. However, based on the results obtained in the context of other viral infections, one may speculate that NPY could have a protective role during HSV-1 infection of the CNS, as such an effect has been reported in the context of retrovirus-induced neurological disease, which shares several traits with HSV-1 neuroinflammation, such as glial cell activation and the production of proinflammatory cytokines in the CNS (186, 187). However, this factor may have detrimental effects during peripheral infections, as a recent study showed that NPY induced a worse course of influenza infection in mice when this neuropeptide was produced by immune cells in the lungs (188).

## Concluding Remarks

An adequate crosstalk and regulation between the nervous system and the immune system surely contributes to the control of tissue homeostasis in healthy conditions, but also disease control during infections. These neuroimmune interactions are governed, at least partially, by soluble factors such as neuropeptides and cytokines. Importantly, in one hand neurons express receptors for immune-derived soluble mediators that can modulate neuronal circuits, and on the other, immune cells have receptors for neuropeptides that can influence their hematopoiesis, priming, migration, and cytokine production, among others.

Furthermore, nerves that innervate the skin can regulate the activity of innate and adaptive immune cells, as well as skin immunity, which will likely play important roles in fighting pathogens that infect epithelial cells and enter the body through this route, as is the case for HSV-1. Remarkably, relatively little is known about how this virus is able to successfully persist in host neurons and then reactivate, causing sporadic or periodic reactivation episodes. Interestingly, important roles have been attributed to immune cells during these viral processes, which likely relates to the vicinity these cells have with HSV-1-infected skin cells and neurons in the TG. However, more studies on which neuropeptides are locally produced in the TGs and how they affect infiltrating immune cells and viral latency, as well as HSV-1 reactivation are needed.

Given the impact of HSV-1 in human health given by the uncomfortable skin lesions it produces, but also by the permanent damage it can elicit in the cornea and brain, it is of great importance to understand those host components that control, facilitate or modulate viral infection and latency. A better understanding of the various processes that modulate the interactions between the nervous system, the skin and immune components will likely contribute at identifying and developing new therapeutic strategies against HSV-1 infections and limit the burden caused by this virus on human health.

## Author Contributions

All authors reviewed the manuscript. All authors contributed to the article and approved the submitted version.

## Funding

This work was funded by ANID - Millenium Science Initiative Program - ICN09_016 : Millenium Institute on Immunology and Immunotherapy (ICN09_016; P09/016-F) and FONDECYT grant #1190864 from the Agencia Nacional de Investigación y Desarrollo (ANID). This work was also supported by the Regional Government of Antofagasta through the Innovation Fund for Competitiveness FIC-R 2017 (BIP Code: 30488811-0). MF is an ANID fellow #21191390.

## Conflict of Interest

The authors declare that the research was conducted in the absence of any commercial or financial relationships that could be construed as a potential conflict of interest.
